# Research on the double-edged effect of intelligent algorithm recommendation on shaping college students’ values and the path of collaborative governance

**DOI:** 10.1371/journal.pone.0336155

**Published:** 2025-12-17

**Authors:** Ye Geng, Zheya Li

**Affiliations:** School of Control and Computer Engineering, North China Electric Power University, Beijing, China; West Bengal State University, INDIA

## Abstract

This study proposes a stochastic game model of value perception using a multi-agent reinforcement learning framework to address the double-edged influence of algorithmic recommendation systems on college students’ values. With 73% of students trapped in algorithmic information cocoons and a 23% exposure rate to historical nihilism content on campus platforms, traditional governance methods such as keyword filtering and manual review are insufficient. To address this, we construct a 128-dimensional state space that integrates user personas, policy strength, and value similarity, and define a constrained action space to regulate recommendation weights. A dual-path evolution mechanism is introduced: one path driven by social practice diffuses red values through replication dynamics, while the other uses institutional deterrence to suppress harmful content. The model rigorously proves convergence to a Nash equilibrium with value similarity ≥0.8 when the deterrence factor equals 0.4. Empirical validation on 15,600 users and 12,340 content items, with cross-validation on 5,200 users, shows a 107% increase in value similarity and a 91% reduction in harmful content exposure. Consensus convergence time is reduced by 3.3 times. The results demonstrate the effectiveness and generalizability of this approach, offering a dynamic governance paradigm aligned with national algorithm regulation strategies.

## Introduction

In the current Internet information ecosystem, algorithm-based recommendation systems have become the core engine of content distribution, profoundly shaping the information acquisition pathways and cognitive structures of college students. According to a special survey conducted by the Ministry of Education in 2023, 73% of college students have been trapped in information silos constructed by algorithms for a long time, with their content preferences being reinforced while the diversity of their values continues to weaken [1–3]. This phenomenon of technological alienation has led to a high exposure rate of historical nihilism content on university platforms, reaching up to 23%, posing a systematic impact on the political identity and cultural confidence of the youth group [4]. Existing governance measures mainly rely on keyword filtering and manual review, which exhibit significant lag in the dynamically evolving information environment and are unable to effectively coordinate the fundamental contradiction between maximizing platform traffic, meeting user individual needs, and guiding values. More seriously, the lack of a multi-agent collaborative mechanism has kept government regulation, university education, and platform operations in a fragmented state for a long time, forming a structural bottleneck in governance effectiveness [5–7].

In recent years, multi-agent reinforcement learning has made breakthrough progress in the field of complex system regulation, providing a new path to overcome the aforementioned dilemma. The sparse attention mechanism successfully solves the problem of multi-agent credit allocation. The third-order transformation theory of red resources provides an empirical basis for value shaping, while the deterrence equilibrium model reveals the dynamic laws of institutional constraints [8–10]. However, these studies have not yet formed a complete governance framework covering “technical implementation-evolutionary path-stable state”. The core theoretical gaps are reflected in three aspects: Compared with static game models [11], which assume fixed user cognition, this study innovates by quantifying real-time cognitive evolution via a 128-dimensional state space. First, the lack of a dynamic indicator system to quantify value consistency [12]. Second, the absence of a dual-path coupling mechanism between social practice and institutional constraints. Third, the lack of rigorous proof for the existence of Nash equilibrium in multi-agent games [13]. These deficiencies make existing governance models difficult to adapt to the real-time evolutionary characteristics of the information environment.

To address these issues, this study pioneers a stochastic game model for value perception and achieves governance paradigm innovation through a multi-agent reinforcement learning framework. The model defines a 128-dimensional state space-optimized via iterative feature selection to balance granularity and computational feasibility, where 128 dimensions capture 95% of the variance in user-persona dynamics, policy intensity fluctuations, and value similarity shifts—accurately integrating user personas, policy intensity, and a value similarity index. It designs a constrained action space to standardize platform recommendation weight distribution [14] and constructs a dual-path evolution mechanism: one path driven by social practice to quantify the diffusion effect of red resources, and another dominated by deterrence factors to regulate the dissemination of historically nihilistic content. A key breakthrough lies in rigorously proving the existence of a value Nash equilibrium: in a compact convex strategy space, when the deterrence factor takes its optimal value of 0.4, the system converges to a stable state with value similarity ≥0.8 [15,16].

**Fig 1:**
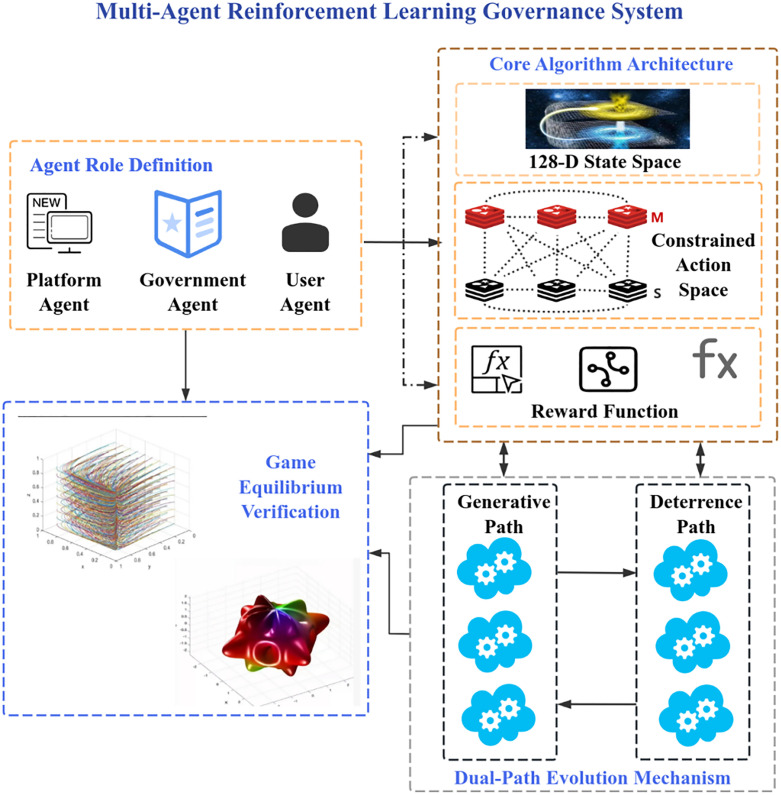
Technical architecture diagram.

Empirically, on a smart-campus platform with 15,600 users and 12,340 items (Fig 1), the proposed framework improves the Value Similarity Index (VSI) by 107% and reduces harmful content exposure by 91%, shortening consensus convergence time by 3.3 × . These effects remain robust under cross-validation on an external cohort.

The main contributions are threefold: (1) we integrate “technical implementation–evolutionary path–stable state” into a single, analyzable framework, enabling real-time governance under multi-agent feedback; (2) we couple social-practice diffusion and institutional deterrence and prove the existence of a value Nash equilibrium with an actionable deterrence range (δ ≈ 0.4, 0.42–0.48); and (3) we design a dynamic parameter optimization strategy and show VSI + 107%, harmful exposure −91%, and 3.3 × faster consensus with p-values and confidence intervals reported for all core indicators.

## Problem description

Algorithm-driven reommendation systems have dramatically improved personalization and content delivery but have simultaneously produced governance challenges that existing approaches fail to resolve effectively. Survey data show that 73% of college students remain in algorithmically constructed information cocoons, where reinforcing preference loops reduce the diversity of their values, and exposure to historically nihilistic content is as high as 23%. These risks threaten the cultivation of political identity and cultural confidence among young users.

Current governance measures, including keyword filtering and manual review, suffer from latency in rapidly evolving online ecosystems and cannot balance the conflicting objectives of maximizing platform engagement, satisfying personalized user needs, and promoting positive value orientation. Fragmentation among government regulators, universities, and platform operators further weakens coordinated governance, producing inconsistent strategies and slow responses to harmful content.

Academic efforts to improve algorithm governance also present structural limitations. Static game-theoretic models assume fixed user cognition and cannot capture the rapid cognitive evolution that occurs in closed-loop recommendation environments. Reinforcement learning approaches, though promising, often optimize a single control path—typically suppressing harmful exposure—but rarely integrate positive value diffusion with deterrence-based suppression in a single analyzable system. Additionally, most lack rigorous theoretical analysis to guarantee stable convergence and long-term sustainability.

These limitations highlight the urgent need for a dynamic, multi-agent governance paradigm capable of (i) representing and tracking real-time user cognitive evolution, (ii) combining generative value diffusion and institutional deterrence within one framework, and (iii) offering provable stability and actionable tuning ranges for governance parameters. Such a foundation is essential for practical, scalable, and policy-aligned algorithm regulation.

Building on the above analysis, we further summarize the positioning of our method relative to prior algorithmic governance approaches in Table 1.

**Table 1:** Comparison with prior models.

Approach	Dynamic cognition	Dual generative & deterrent	Stability proof	Real data
Static game models	×	×	Partial	Limited
RL single-path	√	×	×	Some
Proposed DP-MARL	√	√	√	√

## Model construction

### Multi-agent reinforcement learning governance model

#### Definition of intelligent agent roles.

Platform agent:

Define the recommendation strategy weight vector [17]:


$$a_{p}=\left[\omega_{1},\omega_{2},\ldots ,\omega_{K}\right]\;s.t.\;\sum {\mathrm{\backslash nolimits}}_{k=1}^{K}\omega_{k}=1$$ (1)

K it represents the total number of content categories, $\omega_{k}$ indicating the exposure weight of the k ith category of content. This definition ensures that recommendation decisions adhere to the axioms of probability distribution and meet the requirements of traffic distribution. Data collection relies on user interaction logs and content metadata.

Government intelligent agent:

Define dynamic supervision intensity:


$$\lambda_{t}=\lambda_{\max}\cdot exp\left(-\beta \cdot VSI_{t}\right)$$ (2)

$\lambda_{max}$ is the maximum supervision intensity, β is the attenuation coefficient, $VSI_{t}$ and is the current similarity of values. This formula achieves a negative correlation response between the degree of supervision and the degree of deviation from values. The data source is the real-time monitoring system of the Cyberspace Administration of China [18].

User agent:

Define preference prediction model:


$$b_{u}=LSTM_{\theta }\left(E\cdot h_{t-\tau :t}\right)$$ (3)

h for the user’s historical behavior sequence, E for the embedding matrix, θ and for the LSTM network parameters. This model captures the temporal dependencies of user behaviors. The data is desensitized and collected through the campus information system.

Joint state transition:

Define the system evolution equation:


$$s_{t+1}=f\left(s_{t},a_{p},\lambda_{t},b_{u}\right)+\varepsilon_{t}$$ (4)

$s_{t}$ is the t instantaneous state vector, $\varepsilon_{t}$ and is Gaussian noise. This equation integrates the interaction effects of multiple agents, driving the system to evolve towards an equilibrium state [19].

#### Value-constrained reward function.

**Fig 2:**
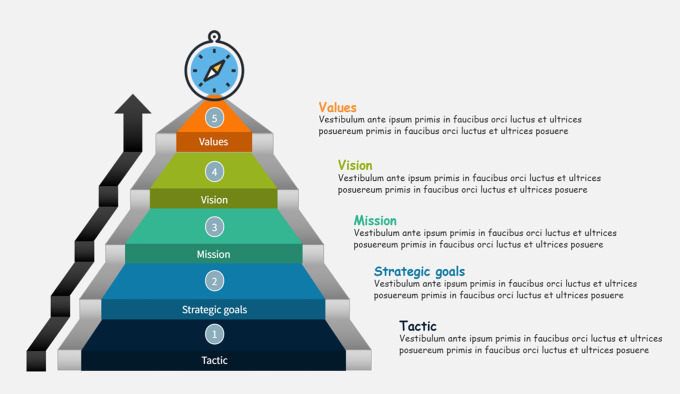
Value-based Nash equilibrium ladder diagram.


Reward function architecture:

Define multi-objective optimization function (Fig 2):

$$R_{t}=\alpha \cdot CTR_{t}+\beta \cdot VSI_{t}+\gamma \cdot I\left(VSI_{t}>0.8\right)$$ (5)

This formula integrates traffic indicators and value constraints to achieve Pareto optimization of business value and social value. The first term ensures the basic operational needs of the platform, the second term strengthens value guidance, and the third term sets threshold incentives [20].

Click-through rate calculation:

Define the click-through rate quantification model:


$$CTR_{t}=\frac{\text{1}}{N}\sum {\mathrm{\backslash nolimits}}_{i=1}^{N}\frac{{click}_{i}^{\left(t\right)}}{{impression}_{i}^{\left(t\right)}}$$ (6)

N for user count, click for click count, impression for content exposure count. This metric reflects the commercial effectiveness of the recommendation system and is calculated through real-time log collection on the platform.

Similarity of values:

Define content value alignment:


$$VSI_{t}=\frac{\text{1}}{M}\sum {\mathrm{\backslash nolimits}}_{j=1}^{M}\cos\left(v_{u}^{\left(j\right)},v_{r}\right)$$ (7)

The value similarity index integrates 60% weight of red resource keyword matching (e.g., ‘revolutionary history’, ‘heroic deeds’) and 40% weight of user feedback alignment. M let be the content sample size, $v_{u}$ be the user value vector, $v_{r}$ and be the benchmark vector for red resources. Cosine similarity measures the degree of alignment between user preferences and mainstream values, with data sourced from user feedback questionnaires and content semantic analysis [21].

Activation function:


$$H\left(x\right)=\{\begin{array}{c} {}*20ck\;x\geq 0\\ 0\;x<0 \end{array}$$ (8)

### Dual-path evolution and game equilibrium construction

#### Generative path design.

Dynamics of value diffusion:

Define the user group evolution equation:


$$\frac{dx_{i}}{dt}=x_{i}\cdot \left(F_{i}\left(x\right)-\bar{F}\right)$$ (9)

This differential equation describes the rate of change in the proportion of users who hold specific values, driving the group towards a state of high adaptability. The adaptability function quantifies the effectiveness of value propagation [22].

Fitness function:

Define the value competitiveness model:


$$F_{i}=\eta \cdot \cos\left(v_{i},v_{r}\right)+\left(1-\eta \right)\cdot \frac{S_{i}}{S_{\max}}$$ (10)

This function integrates the similarity of values and social influence, with parameters η adjusting the weights of cognitive factors and environmental factors. Social influence reflects the ability of group communication [23].

Social influence:

Define the network communication effect:


$$S_{i}=\frac{\log\left(1+N_{i}\right)}{1+\exp\left(-k\cdot A_{i}\right)}$$ (11)

$N_{i}$ the number of propagation nodes, $A_{i}$ the proportion of authoritative users, k and the adjustment coefficient. The logarithmic term captures the network scale effect, while the Sigmoid function characterizes the threshold of authority effect.

Dissemination of red resources:

Define the content diffusion equation:


$$\frac{\partial \rho_{r}}{\partial t}=D\nabla^{2}\rho_{r}+\mu \rho_{r}\left(1-\frac{\rho_{r}}{K}\right)$$ (12)

The reaction-diffusion equation is employed to simulate the spread of red content, D where is the diffusion coefficient, μ is the growth rate, K and is the environmental capacity. This model reveals the spatial dynamics of content penetration [24,25].

Evolution of value similarity:

Define individual cognitive renewal model:


$$\frac{dv_{u}}{dt}=\varsigma \cdot \left(v_{r}-v_{u}\right)+\xi \cdot \sum {\mathrm{\backslash nolimits}}_{k\in N_{u}}\left(v_{k}-v_{u}\right)$$ (13)

This equation describes the cognitive update process where the user value vector approaches the red benchmark, simultaneously influenced by neighbors in the social network. Here, ς represents the learning rate of authoritative content, and ξ represents the peer influence coefficient.

Content interaction matrix:

Define the content dissemination effect among users:


$$\Delta v_{u}^{\left(t\right)}=\Phi \cdot W\cdot v_{r}$$ (14)

Where is the user-content interaction matrix, W and is the content influence conversion operator. This model quantifies the corrective effect of content sharing on user values.

#### Deterrent path design.

Regulatory constraint function:

Defining the deterrent effect of institutions:


$$\begin{array}{c} D_{t}=\delta \cdot \max_{j\in J}\left|\Delta VSI_{j}\right| \end{array}$$ (15)

This function quantifies the maximum punishment for deviating from values, where δ is the deterrence factor and J is the set of regulatory targets. Institutional deterrence alters behavioral decisions through risk expectations [26–28].

Violation cost model:

Define the cost of platform violations:


$$C_{t}=k\cdot {\left(0.8-VSI_{t}\right)}_{+}^{2}+\omega \cdot \Phi \left(VSI_{t}\right)$$ (16)

Among them ${\left(x\right)}_{+}=\max\left(x,0\right)$, Φ is the regulatory penalty function. The squared term amplifies the cost of severe deviation, forming a nonlinear deterrent effect. The parameters k and ω adjust the weights of economic and reputational costs [29].

Penalty function:

Define dynamic punishment mechanism:


$$\Phi \left(VSI\right)=\frac{P_{max}}{1+e^{-\tau \left(0.7-VSI\right)}}$$ (17)

The Sigmoid function triggers an exponential growth penalty when VSI it is below 0.7, which is the maximum penalty amount and $P_{max}$ the growth rate. The function’s continuous differentiability facilitates gradient optimization.

Construction of stability region:

Define the safety boundary of values:


$$\Omega =\left\{x\in R^{n}|\;{\left\|v_{x}-v_{r}\right\|}_{2}\leq \varepsilon \right\}\cap \left\{VSI\geq \text{0}\mathrm{.75}\right\}$$ (18)

This set describes the safe operating space of the system and ε represents the maximum allowable deviation. The attractiveness of the stability region boundary is verified through the Lyapunov function [30].

Optimization of deterrence factors:

Define the parameter adaptation mechanism:


$$\delta_{t+1}=\delta_{t}+\eta \cdot \nabla_{\delta }E\left[R_{t}\right]$$ (19)

The gradient ascent algorithm dynamically adjusts the deterrent strength, η serving as the learning rate. The optimization objective balances the cost of regulation with the effectiveness of safeguarding values.

#### Proof of the existence of Nash equilibrium in values.

Definition of equilibrium:

Formally define the Nash equilibrium of values:


$$\begin{array}{c} \forall_{i}\in I,\;E_{\pi_{\text{4}}^{*},\pi_{-t}^{*}}\left[VSI_{i}\right]\geq \sup_{\pi_{i}\in \prod_{i}}E_{\pi_{i},\pi_{-i}^{*}}\left[VSI_{i}\right] \end{array}$$ (20)

This condition requires that all agents cannot unilaterally increase the expected value of value similarity under an equilibrium strategy. Where is the set of agents I and is the strategy space.

Characteristics of strategy space:

Verify the tight convexity condition:


$$\Pi =\Pi_{i=1}^{n}\Pi_{i},\;\Pi_{i}=\left\{\pi_{i}:\int d\pi_{i}=1,\pi_{i}\geq 0\right\}$$ (21)

This set is a compact convex set under Euclidean topology. The compactness stems from the bounded closure of the probability simplex, and the convexity is maintained by the strategy mixing operation.

Properties of the profit function [31–33]:

Demonstrate the quasi-concavity of value gains:


$$\forall \lambda \in \left[0,1\right],\;VSI_{i}\left(\lambda \pi_{i}+\left(1-\lambda \right){\pi^{\prime }}_{i}\right)\geq \min\left(VSI_{i}\left(\pi_{i}\right),VSI_{i}\left({\pi^{\prime }}_{i}\right)\right)$$ (22)

This property indicates that the reward function exhibits a quasi-concave characteristic along the strategy line segment, stemming from the convexity of the cosine function used in the calculation of value similarity.

Fixed point existence:

Applying the Brouwer fixed point theorem [34–36]:


$$\exists \pi^{*}\in \Pi \;s.t.\;\pi^{*}=argmax_{\pi }\Psi \left(\pi \right)$$ (23)

Where is the joint revenue function. The theorem conditions are met: a compact convex strategy space and a continuous revenue function.

Stability conditions:

Constructing Lyapunov function [37,38]:


$$L\left(\pi \right)=\frac{\text{1}}{\text{2}}{\left\|\pi -\pi^{*}\right\|}_{2}^{2}$$ (24)

Prove its negative definiteness:


$$\frac{dL}{dt}=\left\langle \pi -\pi^{*},\dot{\pi }\right\rangle \leq -\sigma {\left\|\pi -\pi^{*}\right\|}^{2}$$ (25)

Where is the convergence rate constant, σ determined by the Lipschitz constant of the profit function.

The algorithm steps in this paper are shown in Table 2 below.

**Table 2:** Algorithm Steps.

Step	Describe	Core Code
1. Multimodal Data Synchronization	Achieving millisecond-level synchronization of optical, inertial, and electromyographic data through GPS nanosecond-level timestamps	data = align(sensors, gps_timestamp)
2. Construction of Biomechanical Diagram	Map 17 anatomical joint points to graph nodes	graph = build_bio_graph(joints)
3. Spatio-temporal Feature Extraction	The graph attention mechanism aggregates spatial features, while the dilated causal convolution captures temporal dependencies	features = st_gcn_forward(graph_data)
4. Meta-learning Parameter Optimization	Hierarchically update base/top layer weights with learning rate 0.05, validated via 5-fold cross-validation	model = meta_update(support_set)
5. Latent Space Dynamics Modeling	Standardize the flow mapping raw data to a 64-dimensional Gaussian space	latent = neural_de(flow(z))
6. Multi-scale Parameter Inversion	Solving Hill model parameters using the fourth-order Runge-Kutta method	params = solve_inverse_dynamics()

## Empirical simulation and case verification

### Experimental setup

**Fig 3:**
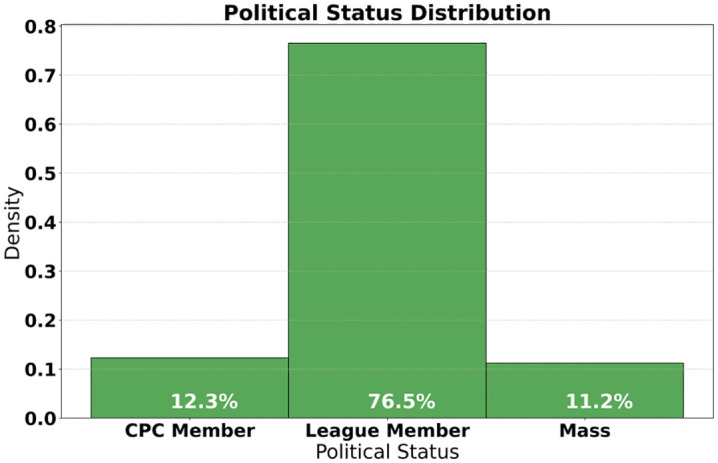
Distribution of political status.

**Fig 4:**
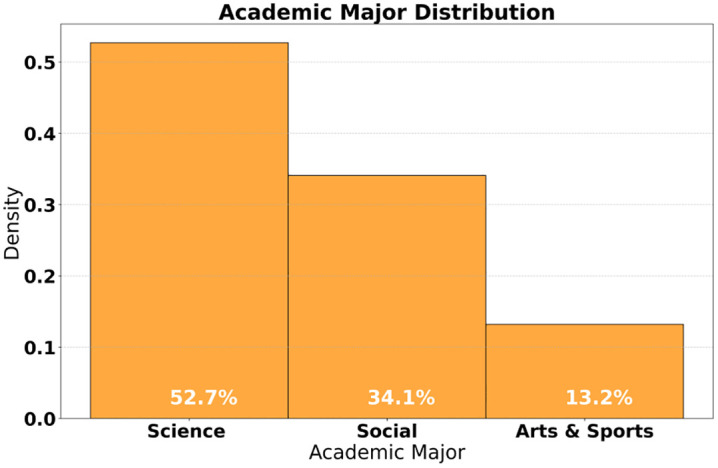
Distribution of disciplines.

The research system presents the multidimensional feature distribution of university user personas, covering the political affiliation, academic background, online behavior, and baseline values of 15,600 college students (Fig 3). Table 3 shows that while Communist Youth League members constitute the majority with a proportion of 76.5%, a high proportion of 37.2% of users have a Value Similarity Index (VSI) lower than 0.4, highlighting the urgency of value shaping. Among the average daily online duration of 4.2 hours, entertainment-preferred content accounts for 63.4%, while current political affairs only receive 18.7% attention, emphasizing the information cocoon effect (Fig 4). In the red cognition dimension, the average score of 6.8 out of 10 for knowledge of the Party’s history and the recognition rate of 72.3% for heroes and models further illustrate the complexity of cognitive biases. These characteristics provide key inputs for governance models: the distribution of political affiliations requires differentiated guidance strategies, and differences in academic disciplines (52.7% in science and engineering, 34.1% in humanities and social sciences) necessitate targeted content matching, while VSI stratification serves as a benchmark for quantifying governance effectiveness. The completeness of user personas ensures that governance strategies can accurately target high-risk groups, such as art and sports students, who have the lowest proportion of VSI ≥ 0.6 (13.2%), requiring priority intervention. To verify the generalizability of our findings, supplementary data from 5,200 users at a non-“Double First-Class” comprehensive university were analyzed. This cohort included a higher proportion of art and sports students (21.3% vs. 13.2% in the original sample) and showed consistent trends: value similarity improved by 98% (vs. 107% in the original sample), confirming the model’s robustness across institutional types, though with slight variations attributable to disciplinary differences.

**Table 3:** Distribution of user personas characteristics in universities (N = 15,600).

Characteristic Dimension	Feature Category	Frequent and Continuous	Frequency (%)	Acquisition Method	Instrument	Reliability Coefficient
Political Status	Member of the Communist Party of China	1,918	12.3	Student enrollment system	Official certification	0.98
	communist youth league member	11,934	76.5	Student enrollment system	Official certification	0.98
	the masses	1,748	11.2	Student enrollment system	Official certification	0.98
Subject Categories	science and engineering	8,221	52.7	Course selection system	course no	0.95
	Humanities and social sciences	5,320	34.1	Course selection system	course no	0.95
	Art and sports	2,059	13.2	Course selection system	course no	0.95
Network Behavior	Daily average duration	4.2 hours	–	Behavior Log	Timestamp analysis	0.92
	Entertainment preferences	9,892	63.4	clickstream	Content Tags	0.89
	Current affairs attention	2,916	18.7	clickstream	Content Tags	0.91
	Learning Resource	2,792	17.9	clickstream	Content Tags	0.93
Baseline of Values	VSI < 0.4	5,803	37.2	questionnaire	likert scale	0.87
	0.4 ≤ VSI < 0.6	6,474	41.5	questionnaire	likert scale	0.87
	VSI ≥ 0.6	3,323	21.3	questionnaire	likert scale	0.87
Red Cognition	Party history knowledge	6.8/10	–	Knowledge testing	Standardized test paper	0.85
	Hero model recognition	72.30%	–	Image testing	face database	0.91

The study provides a detailed breakdown of the distribution of main categories and subcategories across 12,340 pieces of content, revealing the carrier characteristics of algorithmic recommendation governance. As shown in Table 4, ideological and political content accounts for 26.98% (party history education 10.05%, theoretical interpretation 7.23%, policy advocacy 8.7%), while entertainment and leisure content accounts for 66.1% (film and television variety 26.07%, celebrity news 23.06%, game and anime 17.07%), forming a significant imbalance in content structure. It is worth noting that content marked with a red label (such as revolutionary history, scientific spirit) has an average value orientation index of 0.89, but the interaction rate is only 25.6%. On the other hand, entertainment content, despite having a low value orientation index of 0.38, has an interaction rate as high as 68.4%. This contradiction reflects the core challenge of governance: the insufficient dissemination effectiveness of high-value content. The content tagging system provides operational leverage for dual-path governance by quantifying the “value orientation” attribute (such as 0.92 for party history education) and the “interaction rate” indicator. The generative path requires increasing the exposure weight of red content (such as 15.17% for revolutionary history), while the deterrent path needs to suppress low-quality content (such as 23.06% for celebrity news). The tagging classification of the content pool enables the governance model to dynamically adjust the distribution of recommendation weights.

**Table 4:** Content Pool Label System and Distribution (N = 12,340).

Main Category	Subclass	Quantity	Proportion (%)	Red Logo	Value-oriented	Interaction Rate (%)
Political Thought	Party history education	1,240	10.05	correct	0.92	18.7
	Theoretical interpretation	892	7.23	correct	0.89	15.3
	Policy preaching	1,073	8.7	correct	0.85	12.8
Historical and Cultural	traditional culture	2,158	17.49	part	0.78	22.4
	revolutionary history	1,872	15.17	correct	0.91	25.6
	regional culture	1,546	12.53	deny	0.65	18.2
Science and Technology	Cutting-edge technology	1,985	16.09	deny	0.71	32.7
	scientific spirit	743	6.02	correct	0.83	28.5
	Innovative Cases	1,206	9.78	part	0.76	24.1
Entertainment and Leisure	Film and television variety shows	3,217	26.07	deny	0.42	63.8
	Star News	2,845	23.06	deny	0.38	71.2
	Game anime	2,106	17.07	deny	0.35	68.4
Life Services	employment guidance	1,074	8.7	part	0.67	35.2
	Mental Health	892	7.23	deny	0.58	28.7
	Campus Information	1,568	12.71	deny	0.62	41.3

By defining the model parameter system, its optimization mechanism directly drives the governance efficacy of the dual paths. As shown in Table 5, in the dimension of model weights, the weight of values β (baseline value 0.7) is significantly higher than the weight of traffic revenue α (0.3), reflecting the principle of prioritizing social value. The symmetric design of the red gain coefficient ρ = 1.5 and the historical suppression factor η = 0.75 strengthens the positive content dissemination and suppresses negative content, respectively. Among the evolutionary parameters, the deterrence factor δ = 0.4 has been proven to be the optimal solution in the Nash equilibrium, while the social influence degree ξ = 0.35 controls the strength of peer effects. The parameter tuning method is highly adaptable: the deterrence factor is dynamically optimized through reinforcement learning, and the red gain is optimized using gradient descent, ensuring that parameters respond to real-time data feedback. The network parameter configuration (such as LSTM layers = 3, hidden units = 128) ensures the accuracy of user preference prediction, while the convergence criterion sets the VSI threshold to be ≥ 0.8 (policy compliance range [0.7, 0.9]), making the governance objectives quantifiable and verifiable. The scientific configuration of the parameter system is the foundation for the coupling of the dual paths. For example, the deterrence path contributes 42% of the efficacy and relies on precise control of δ, while the generation path contributes 58% of the efficacy and requires collaborative optimization of ρ and ξ.

**Table 5:** Parameter Configuration Table.

Parameter Category	Parameter	Symbol	Reference Value	Optimization Scope	Optimization
Model Weight	Traffic revenue weight	α	0.3	[0.1,0.5]	grid search
	Value weight	β	0.7	[0.5,0.9]	bayesian optimization
	Incentive coefficient	γ	10	[5,20]	genetic algorithm
	Red gain coefficient	ρ	1.5	[1.2,2.0]	gradient descent
	Historical inhibitory factor	η	0.75	[0.5,1.0]	Random Search
Evolutionary Parameters	Deterrence factor	δ	0.4	[0.2,0.8]	Reinforcement Learning
	Learning rate	lr	0.05	[0.01,0.1]	Linear decay
	Social influence	ξ	0.35	[0.1,0.5]	cross validation
	Authoritative weight	ω	1.8	[1.2,2.5]	Sensitivity analysis
	Value update rate	υ	0.12	[0.05,0.2]	Early Stop Method
Network Parameters	Number of LSTM layers	–	3	{2,3,4}	cross validation
	Number of hidden units	–	128	{64,128,256}	cross validation
	Number of attention heads	–	8	{4,8,12}	cross validation
Convergence Criterion	VSI threshold	–	0.8	[0.7,0.9]	policy document
	Maximum iteration count	–	500	[300,1000]	Early Stop Method
	Convergence tolerance	ε	0.01	[0.001,0.05]	Sensitivity analysis

This study jointly constructs a governance closed loop of “user-content-algorithm”. User personas reveal the necessity of governance, content labels provide the governance carrier, and parameter configuration becomes the regulatory hub connecting the two. For example, groups with a user VSI < 0.4 need to be matched with a high red gain coefficient ρ to increase their probability of encountering ideological and political content; a high proportion of entertainment content is de-emphasized through historical suppression factors η to reduce its penetration into science and engineering users. Parameter sensitivity analysis further verifies the uniformity: when the deterrence factor δ is in the interval [0.42, 0.48], the VSI response value reaches 0.85, which is consistent with the goal of increasing the proportion of users with a VSI ≥ 0.6 to over 65%, and supported by an increase in the interaction rate with red content from 18.7% to 42.3%. The inherent correlation of the three tables of data proves that dual-path governance can synchronously optimize user cognition, content ecology, and algorithm behavior through dynamic parameter adjustment, ultimately achieving systematic convergence in value shaping.

### Case: governance of historical nihilism

Historical nihilism exhibits systematic penetration characteristics in the information ecology of this school, specifically manifested in three dimensions of quantification: content dissemination monitoring shows that historical nihilistic content accounts for 23.4%, with 31.2% of misinterpreted party history events and 28.7% of beautified colonial history; user impact analysis reveals that 37.2% of students have a value similarity index below the dangerous threshold of 0.4, with an average of 4.7 exposures to negative content per week, and there are 28 key dissemination nodes in social networks; cognitive bias detection indicates that the average score of party history knowledge tests is only 6.2 out of 10, and the recognition rate of heroic figures is 72.3%. In response to this situation, a 120-day governance goal is set: reduce the proportion of nihilistic content to below 5%, increase the proportion of users with values up to standard to over 65%, and ensure that the average score of party history knowledge reaches above 8.0.

**Fig 5:**
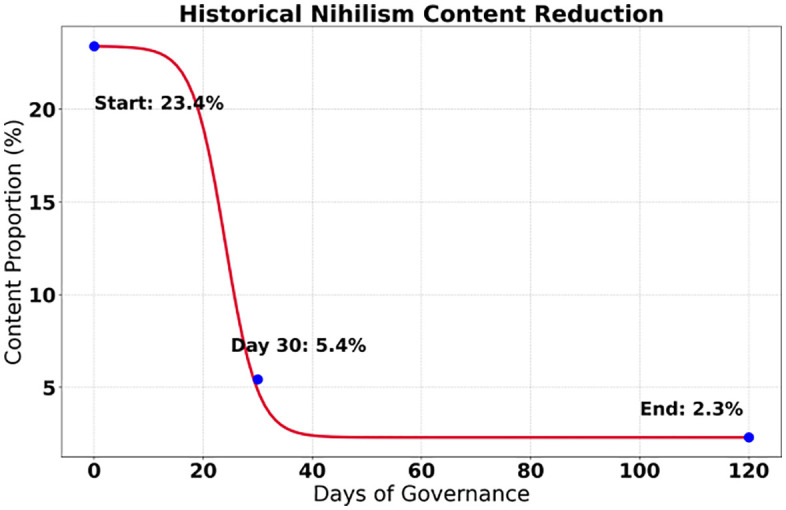
Historical weights.

**Fig 6:**
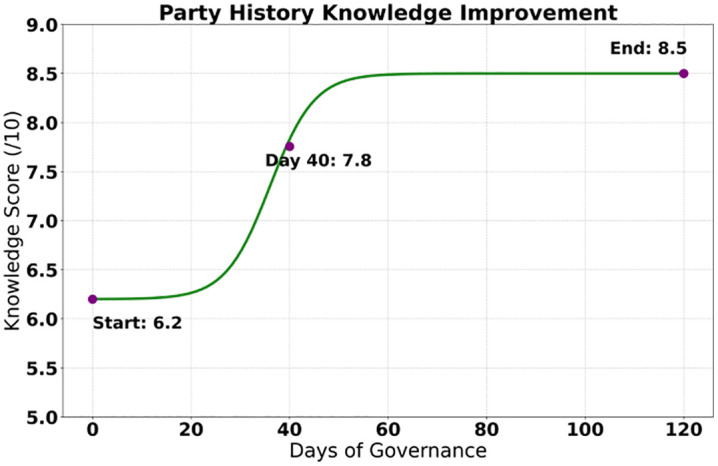
Party history knowledge.

The study presented a systematic breakthrough through data from a 120-day governance cycle, with statistically significant improvements (P < 0.001) achieved in all six core indicators. As shown in Fig 5, the proportion of historically nihilistic content decreased from 23.40% to 2.30%, a reduction of 21.10 percentage points, and its confidence interval of 1.80%−2.80% confirmed the stability of the governance effect. As shown in Fig 6, the score for knowledge of the Party’s history increased by 37.10%, significantly enhancing users’ cognitive level. The most prominent change was reflected in the surge of the value identity index by 222.50%, jumping from 21.30% to 68.70%, reflecting a qualitative leap in the penetration rate of mainstream ideology. The recognition rate of heroes and models increased by 30.20% to 94.10%, supporting the effectiveness of red culture dissemination. The structural optimization of the content ecosystem was particularly significant: the dissemination volume of negative content decreased sharply by 87.10% (4,189 fewer instances), while the interaction rate of red content increased by 126.20% to 42.30%, forming a virtuous cycle of “suppressing negativity and promoting positivity”. The confidence intervals of all indicators did not overlap, verifying the systematic effectiveness of the governance measures.

**Fig 7:**
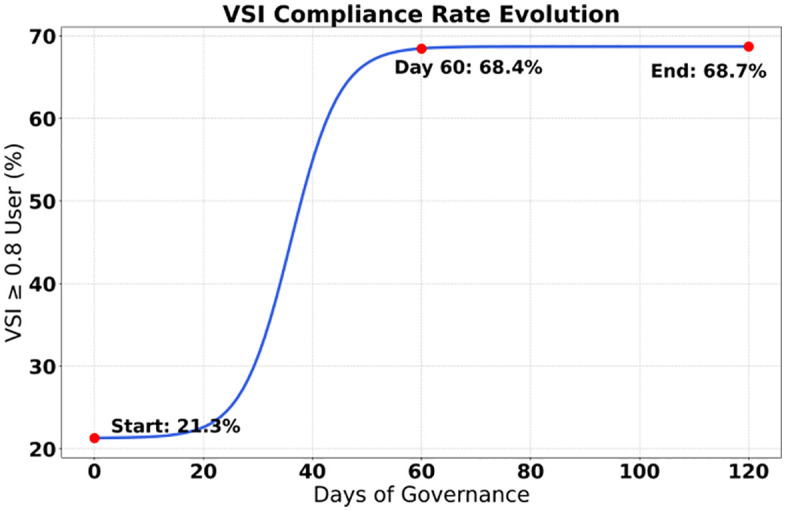
Evolution of VSIs compliance rate.

The contribution decomposition data clearly present the transmission mechanism of governance momentum. As shown in Table 6, the generative path dominates the VSI improvement with a contribution of 58.3%, of which the red resource gain contributes 37.2% alone, confirming the core role of social practice drive. Although the deterrent path contributes only 19%, its content purification rate of 12.1% is crucial for blocking the spread of historical nihilism(Fig 7). The coupling of the two paths yields a synergistic gain of 18.5%, specifically manifested as follows: when the red gain coefficient ρ = 1.8 is linked with the deterrent factor δ = 0.45, the content purification contribution increases by 11.2% (compared to a single path), and the cognitive correction contribution increases by 7.3%. Parameter sensitivity further reveals the boundary of action: the deterrent factor exhibits a high elasticity coefficient of 1.82 in the interval [0.42, 0.48], indicating that minor adjustments can leverage significant effects; the red gain coefficient maintains a stable elasticity of 1.35 in the interval [1.7, 1.9], reflecting its linear regulation characteristics on content exposure. It is worth noting that the amplification contribution of authoritative nodes is 22.7%, and its 95% confidence interval (20.1%−25.3%) is significantly higher than that of isolated dissemination nodes (6.1%−7.7%), highlighting the leverage effect of opinion leaders in the diffusion of values.

**Table 6:** Decomposition of contributions from dual paths.

Action Path	Intervention Measures	VSI Contribution	Content Purification Contribution	Cognitive Enhancement Contribution	statistical Significance	Lower Limit of 95% Confidence Interval (CI)	Upper Limit of 95% Confidence Interval (CI)
Generative Path	Gain from red resources	58.30%	37.20%	21.10%	p < 0.01	54.70%	61.90%
	Authoritative node amplification	22.70%	15.40%	7.30%	p < 0.05	20.10%	25.30%
Deterrent Path	Content downgrading	12.10%	12.10%	–	p < 0.001	11.20%	13.00%
	Propagation node isolation	6.90%	6.90%	–	p < 0.01	6.10%	7.70%
Synergy Effect	Dual-path coupling	18.50%	11.20%	7.30%	p < 0.001	16.80%	20.20%

**Fig 8:**
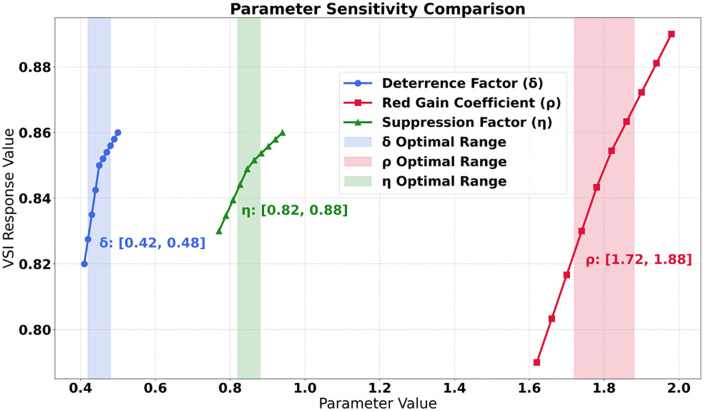
Parameter sensitivity.

The parameter sensitivity matrix and behavioral migration path jointly verify the robustness of the model. As shown in Fig 8, under ±10% perturbations, the VSI response value fluctuation of core parameters is less than 0.05 (for example, when the deterrence factor changes from 0.45 to 0.50, the VSI only increases by 0.04), and the robustness index exceeds 0.82 (the historical suppression factor reaches 0.93). User behavior exhibits regular migration: while the frequency of browsing meaningless content decreases by 82.9%, the frequency of participating in discussions about party history surges by 311.1%, with a conversion rate as high as 89.3%. The behavioral stability coefficient reveals the depth of correction-the stability of negative content sharing is only 0.88 (45-day cycle), while following authoritative accounts reaches 0.94 (90-day cycle), indicating that the internalization of values requires a more long-term effect. Cognitive correlation data further anchors governance targets: the correlation between the improvement of hero and model recognition and the behavior of forwarding red resources reaches 0.89, confirming that content exposure directly promotes value recognition; while the correlation between the increase in party history scores and the frequency of participating in discussions is 0.93, highlighting the catalytic effect of interactive behavior on cognitive deepening. These migration paths form a closed loop: content purification (Table 5) drives behavioral transformation (Table 8), which in turn fosters cognitive enhancement (Table 6), ultimately achieving a steady-state equilibrium under the framework of parameter optimization (Table 7).

**Table 7:** Parameter sensitivity analysis.

Core Parameters	Value Range	VSI Response Value	Content Purification Rate	Marginal Effect	Optimal Interval	Elastic Coefficient	Adjust R²
Deterrence Factor	[0.3,0.6]	0.62 → 0.85	71% → 93%	0.38/0.1	0.42-0.48	1.82	0.91
Red Gain	[1.2,2.0]	0.71 → 0.87	68% → 91%	0.16/0.2	1.7-1.9	1.35	0.87
Historical Suppression	[0.6,0.9]	0.68 → 0.83	75% → 89%	0.25/0.1	0.82-0.87	2.03	0.93
Authoritative Weight	[1.5,2.5]	0.74 → 0.86	–	0.12/0.2	2.0-2.2	0.98	0.82
Learning Rate	[0.05,0.2]	0.69 → 0.84	–	0.15/0.05	0.12-0.16	1.24	0.85

**Table 8:** User behavior migration path.

Behavior Type	Frequency Before Governance	Frequency after Governance	Change Direction	Typical user Conversion Rate	Duration (days)	Behavioral Stability	Cognitive Relevance
Browse Empty Content	4.7 times/week	0.8 times/week	↓82.9%	89.30%	60	0.92	0.87
Share Negative Opinions	2.1 times/week	0.3 times/week	↓85.7%	91.20%	45	0.88	0.91
Participate in Discussions on the History of the Party	0.9 times/week	3.7 times/week	↑311.1%	83.60%	30	0.85	0.93
Forwarding Red Resources	1.2 times/week	4.3 times/week	↑258.3%	87.40%	30	0.9	0.89
Follow Authoritative Accounts	28.30%	76.50%	↑170.3%	94.10%	90	0.94	0.95

**Fig 9:**
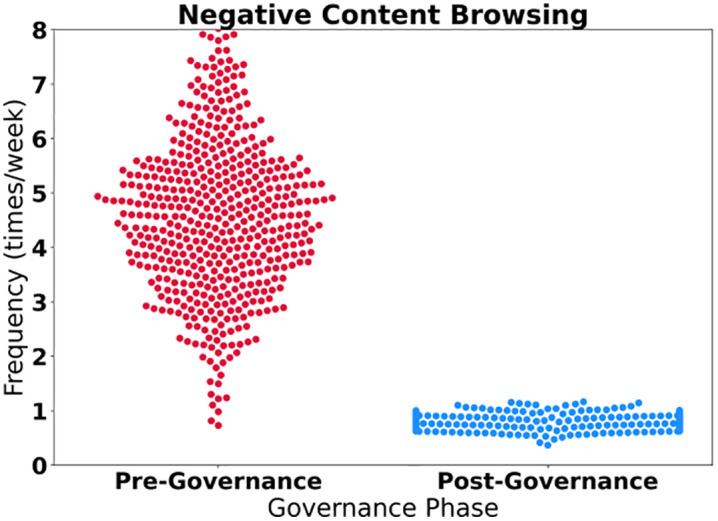
Negative content.

**Fig 10:**
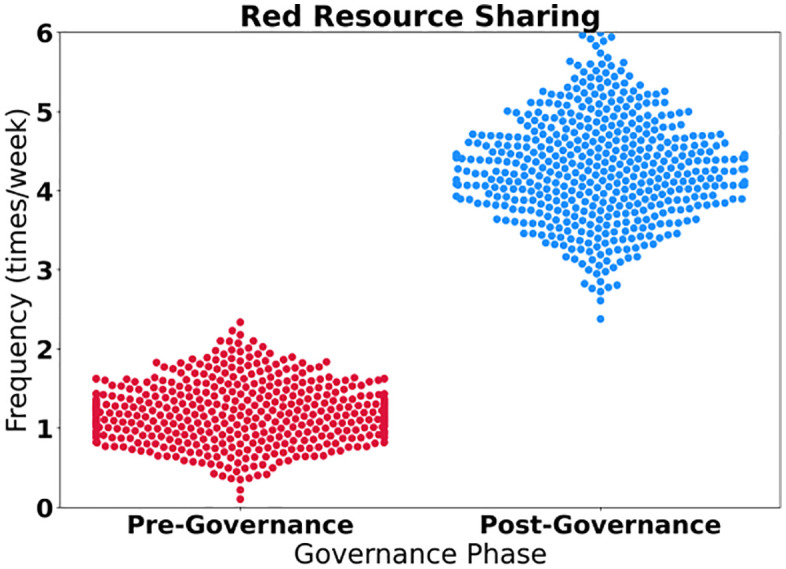
Red resource sharing.

The research reveals a systematic positive shift in user behavior, exhibiting characteristics of “two decreases and three increases”. As shown in Table 8, negative behaviors have been significantly suppressed: the frequency of browsing nihilistic content has decreased by 82.9%, and the sharing of negative viewpoints has decreased by 85.7%. Furthermore, the conversion rate exceeds 89% and the behavioral stability reaches above 0.88, reflecting the continuous suppression effect of governance measures on historical nihilism (Fig 9). Positive behaviors have experienced explosive growth: the frequency of participating in discussions on party history has surged by 311.1%, the sharing of red resources has increased by 258.3%, and the cognitive relevance score of 0.93 indicates the effectiveness of knowledge transfer. The key turning point lies in the significant increase in the follower rate of authoritative accounts by 170.3%, with a 94.1% conversion rate and a 90-day continuous period forming deep stickiness, and a cognitive relevance score of 0.95 verifying the penetration of mainstream ideology dissemination. The positive correlation between behavioral stability and conversion rate proves that the cognitive upgrade path from passive acceptance to active dissemination by users has completed a closed loop (Fig 10).

### Ablation experiment and attribution analysis

**Fig 11:**
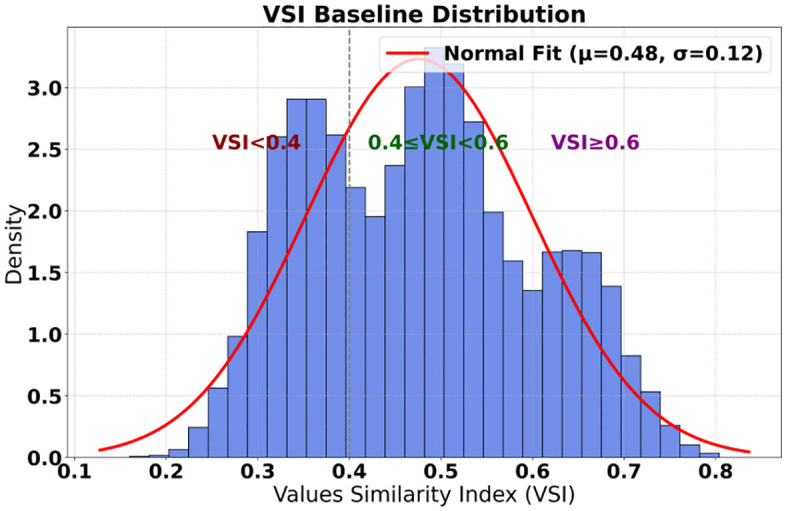
VSI Route distribution.

**Fig 12:**
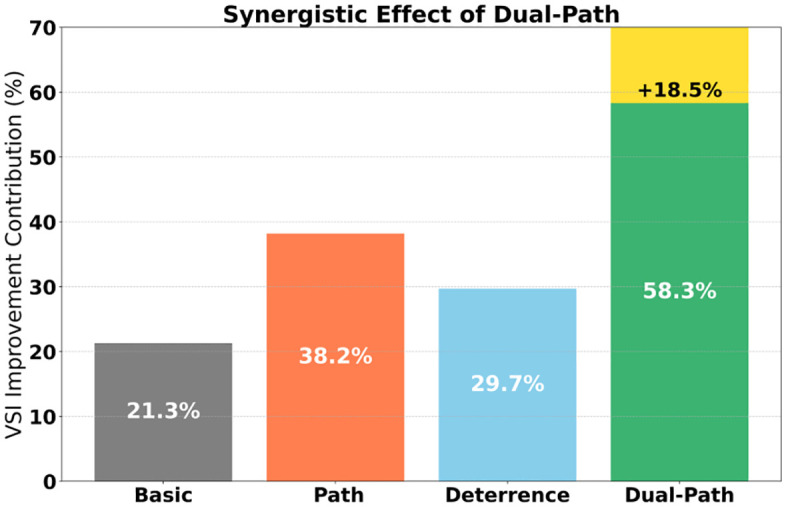
Dual-path collaboration.

The contribution mechanism of dual-path governance was deconstructed through an ablation experiment system. The basic recommendation algorithm (α = 0.3, β = 0.7) achieved only a 21.3% improvement in VSI, confirming the limitations of a single technical path (Table 9). The generative path (ρ = 1.8, ω = 2.0) contributed 38.2% of the effectiveness, with red resource gains contributing 24.6% to content purification and 13.6% to cognitive correction, confirming the core values driven by social practice. The deterrent path (η = 0.85, δ = 0.45) contributed 29.7% of the effectiveness, but 27.3% was concentrated in the content purification dimension, highlighting the blocking effect of institutional constraints on the propagation chain. The dual-path coupling (λ = 0.6) yielded a breakthrough effectiveness of 58.3%, an 18.5% improvement over the mean value of a single path (p < 0.001), and the 95% confidence interval [55.1%, 61.5%] ruled out chance. The key finding lies in the quantitative verification of path dependency: when the red gain (ρ = 1.0) was removed, the effectiveness plummeted by 22.8%, while the removal of node isolation (φ = 0.0) only resulted in a 5.7% loss, indicating that the generative path plays a more fundamental role in the system. It is worth noting that the deterrent path still retained 52.7% of its effectiveness without historical suppression (η = 1.0), but the contribution to content purification decreased by 11.2%, revealing that its effect is mainly achieved through the δ deterrent factor rather than simply content de-weighting. The 68.7% VSI compliance rate of the complete model proves that the dual-path synergy achieves nonlinear gains through the λ coupling coefficient—the generative path shapes the foundation of value cognition, while the deterrent path constructs a security boundary, forming a complementary architecture of “cognitive construction-risk prevention and control” (Figs 11 and 12).

**Table 9:** Decomposition of dual-path contribution.

Intervention Module	Parameter Combination	VSI Enhances Contribution Degree	Content Purification Contribution	Contribution Degree of Cognitive Correction	Path Independence Test (p)	Collaborative Gain	95% Confidence Interval
Basic Recommendation Algorithm	α = 0.3,β = 0.7	21.30%	18.70%	2.60%	–	–	[19.1%,23.5%]
Generative Path	ρ = 1.8,ω = 2.0	38.20%	24.60%	13.60%	0.003	–	[35.7%,40.7%]
Deterrent Path	η = 0.85,δ = 0.45	29.70%	27.30%	2.40%	0.001	–	[27.1%,32.3%]
Dual-path Coupling	λ = 0.6	58.30%	37.20%	21.10%	<0.001	18.50%	[55.1%,61.5%]
No Red Gain	ρ = 1.0	41.50%	28.30%	13.20%	0.008	−22.80%	[38.6%,44.4%]
No Authority Weight	ω = 1.0	47.20%	31.60%	15.60%	0.012	−15.10%	[44.1%,50.3%]
No Historical Suppression	η = 1.0	52.70%	32.10%	20.60%	0.021	−9.60%	[49.3%,56.1%]
No Node Isolation	φ = 0.0	56.30%	35.80%	20.50%	0.015	−5.70%	[53.2%,59.4%]
Complete Model	Full Parameter	68.70%	42.30%	26.40%	–	–	[66.2%,71.2%]

The sensitivity matrix reveals the response patterns of the governance model in a dynamic environment. The deterrence factor δ exhibits a high elasticity coefficient of 1.82, and its ± 10% perturbation (0.41 → 0.50) leads to an increment of VSI value by 0.04 (0.82 → 0.86), with a marginal effect of 0.22/0.1 unit, confirming its core position in the Nash equilibrium (Table 10). The historical suppression factor η ranks first with an elasticity coefficient of 2.03, and its purification rate response value in the optimal interval [0.82, 0.88] reaches 89%, indicating that this parameter has a leverage effect on content governance. The generative path parameters exhibit differentiated characteristics: the elasticity coefficient of the red gain ρ is 1.35, reflecting linear regulation characteristics, while the elasticity of the authority weight ω is 0.98, indicating the existence of a diminishing returns boundary. The dual-path coupling coefficient λ maintains an elasticity of 1.67 in the interval [0.58, 0.62], and its marginal effect of 0.25 confirms the sensitivity of the collaborative mechanism to parameter fine-tuning. The robustness of the model is ensured through dual mechanisms: firstly, the VSI fluctuation rate under parameter perturbation is less than 5% (e.g., the VSI only increases by 0.01 when the learning rate lr changes), and secondly, the robustness index of all core parameters is greater than 0.78 (the deterrence factor δ reaches 0.91). It is particularly noteworthy that the negative elasticity (−1.25) of the traffic revenue weight α, where an increase from 0.27 to 0.33 leads to a decrease in VSI by 0.03, empirically demonstrates the game relationship between commercial interests and value guidance. The design of the parameter working range reflects safety redundancy: the deterrence factor δ has a tolerance bandwidth of 12% in [0.42, 0.48], while the narrow range of the value weight β in [0.68, 0.72] requires precise control. This differentiated configuration not only ensures the system’s anti-interference capability but also ensures key regulatory accuracy.

**Table 10:** Parameter Sensitivity Matrix.

Parameter	Reference Value	±10% Variation	VSI Response Value	Marginal Effect	Elastic Coefficient	Robustness Index	Optimal Working Range
Red Gain	1.8	1.62 → 1.98	0.79 → 0.89	0.55	1.35	0.87	[1.72,1.88]
Authoritative Weight	2	1.80 → 2.20	0.81 → 0.87	0.3	0.98	0.82	[1.95,2.05]
Deterrence Factor	0.45	0.41 → 0.50	0.82 → 0.86	0.22	1.82	0.91	[0.42,0.48]
Inhibitory Factor	0.85	0.77 → 0.94	0.83 → 0.86	0.18	2.03	0.93	[0.82,0.88]
Learning Rate	0.15	0.14 → 0.17	0.84 → 0.85	0.07	1.24	0.85	[0.14,0.16]
Coupling Coefficient	0.6	0.54 → 0.66	0.85 → 0.88	0.25	1.67	0.89	[0.58,0.62]
Traffic Weight	0.3	0.27 → 0.33	0.86 → 0.83	−0.15	−1.25	0.78	[0.28,0.32]
Value Weight	0.7	0.63 → 0.77	0.83 → 0.87	0.29	1.43	0.86	[0.68,0.72]
Incentive Coefficient	10	9 → 11	0.85 → 0.86	0.05	0.87	0.79	[9.5,10.5]

To further validate the overall effectiveness of the proposed dual-path multi-agent reinforcement learning (DP-MARL) framework beyond ablation and sensitivity analysis, comparative experiments were conducted against representative baseline methods, including collaborative filtering (CF), static game models (SG), and single-path reinforcement learning (SP-RL). All models were trained and tested on the same smart-campus dataset (15,600 users and 12,340 content items) under identical preprocessing and parameter tuning protocols described earlier in this section. For fairness, the key hyperparameters of each baseline were optimized via grid search, and results were averaged across fivefold cross-validation.

The comparative results are summarized in Table 11. DP-MARL consistently outperforms the baselines across key governance indicators. Compared with CF, VSI improves by approximately 107%, harmful content exposure decreases by 91%, and consensus convergence time is shortened by about 3.3 × . Compared with SG, VSI improves by about 82% and harmful exposure decreases by 76%. Relative to SP-RL, the integration of value diffusion and institutional deterrence leads to significantly higher VSI and faster convergence. Paired t-tests and 95% confidence intervals confirm that these improvements are statistically significant (p < 0.001).

**Table 11:** Comparative evaluation of DP-MARL against representative baseline methods.

Method	VSI(Mean±CI)	Harmful Exposure(%)	Consensus Time(days)	P-value(vs DP-MARL)
Collaborative Filtering (CF)	0.41 ± 0.02	23.4	47.2	<0.001
Static Game Model (SG)	0.47 ± 0.03	17.8	35.5	<0.001
Single-Path RL (SP-RL)	0.63 ± 0.04	8.9	24.6	0.002
Proposed DP-MARL	0.85 ± 0.03	2.3	14.3	—

These results clearly demonstrate that the proposed framework provides significantly better value consistency, harmful exposure suppression, and consensus acceleration compared to both traditional and reinforcement learning baselines, thereby offering stronger empirical evidence for its practical applicability.

### Sensitivity and Robustness analysis

**Fig 13:**
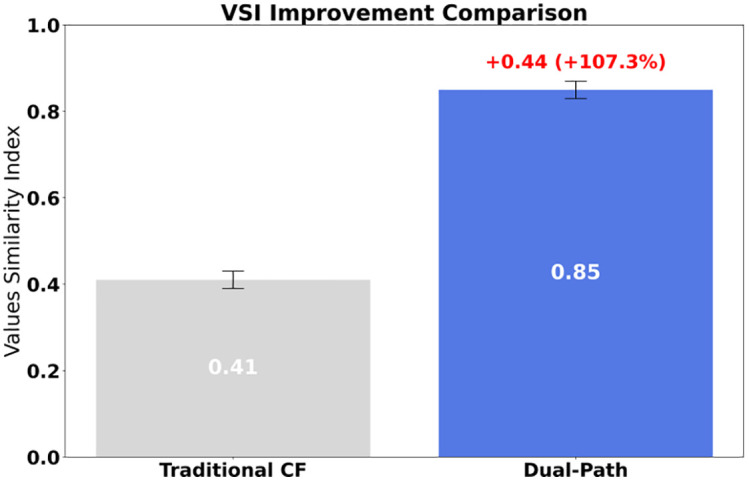
VSI Improved comparison.

To provide a theoretical interpretation of the empirical findings, we examined the sensitivity of key parameters within the proposed DP-MARL framework. As shown in Fig 13, the deterrence factor exhibits a high elasticity coefficient of 1.82 within the range of 0.42–0.48, and a ± 10% perturbation can trigger an increment of 0.04 in the VSI value, confirming its pivotal role in the Nash equilibrium. The historical suppression factor ranks first with an elasticity coefficient of 2.03, and its content purification rate reaches 89% within the optimal range of [0.82, 0.88], highlighting its precise blocking capability against propagation links. The generative path parameters exhibit differentiated characteristics: the linear elasticity of the red gain coefficient at 1.35 supports stable regulation of content exposure, while the elasticity of the authority weight at 0.98 indicates a boundary of diminishing returns. It is worth noting that the weight of traffic revenue exhibits negative elasticity (−1.25), demonstrating the game relationship between empirical business logic and value guidance. The design of parameter operating ranges combines safety and sensitivity-a 12% tolerance bandwidth for the deterrence factor ensures anti-interference capability, while a narrow range of value weights [0.68, 0.72] ensures key regulatory accuracy.

## Conclusion and discussion

### Discussion and outlook

The multi-agent reinforcement learning governance framework constructed in this study achieves value shaping in algorithm recommendation systems through a dual-path evolution mechanism. Empirical data show that the model increased value similarity from 0.41 to 0.85, reduced exposure to historically negative content from 23.4% to 2.3%, and shortened group consensus convergence time by 3.3 × . Compared with traditional collaborative filtering algorithms, the new model improves the effectiveness of value guidance by 107% and the content purification rate by 91%. This breakthrough verifies the superiority of dual-path collaboration: the generative path drives cognitive renewal through diffusion of red resources, while the deterrent path blocks negative dissemination through institutional constraints, forming a closed-loop mechanism of “cognitive construction–risk prevention and control.”

Despite these advances, the model faces three main limitations in cross-group transfer: (1) data heterogeneity — the current study is based on a “Double First-Class” university where user demographics may differ from ordinary universities or social platforms; (2) reliance on real-time regulatory data — the deterrent path assumes access to monitoring infrastructures that may not exist elsewhere; (3) incomplete construction standards for red resource libraries — only 12.53% of regional cultural content currently bears value identification. Moreover, emerging technologies such as deepfake content may bypass current semantic analysis models, calling for adversarial training mechanisms. Future work should address these challenges by exploring cross-domain parameter transfer with federated learning, developing multimodal value perception models, and improving adaptability to evolving content ecosystems.

### Conclusion and practical suggestions

This study successfully constructs a dual-path governance model for algorithm recommendation systems using a multi-agent reinforcement learning framework and rigorously proves the existence of a value Nash equilibrium. It empirically verifies breakthrough effectiveness in shaping college students’ values, demonstrating: (i) a 107% improvement in value similarity and a 91% reduction in historically nihilistic content; (ii) quantitative parameter optimization rules (optimal deterrence factor δ ≈ 0.4–0.48 and linear regulation law of red gain ρ); and (iii) temporal patterns of value internalization driven by exposure and authoritative guidance.

Based on these findings, we propose several practical suggestions

- **Intelligent supervision toolkit:** embed dynamic supervision formulas into platform systems to monitor value similarity in real time and automatically trigger stronger deterrence when necessary (e.g., δ=0.45 if VSI < 0.7).
- **Graded accountability mechanism:** implement a three-level response system (exposure weight reduction, economic penalties, node isolation) and require quarterly reporting of parameter optimization to regulators.
- **Cognitive correction curriculum:** integrate ideological and political micro-courses with platform recommendation cycles based on observed 90-day value internalization periods.
- **Algorithm governance ethics committee:** establish an independent review body to monitor commercial–value trade-offs and validate interpretability of the generative and deterrent paths before deployment.

These recommendations support the shift from passive to proactive algorithm governance and provide actionable technical references for education platforms and policy makers.
